# The muscle protein synthetic response following corn protein ingestion does not differ from milk protein in healthy, young adults

**DOI:** 10.1007/s00726-023-03377-z

**Published:** 2024-02-05

**Authors:** Philippe J. M. Pinckaers, Michelle E. G. Weijzen, Lisanne H. P. Houben, Antoine H. Zorenc, Imre W. K. Kouw, Lisette C. P. G. M. de Groot, Lex. B. Verdijk, Tim Snijders, Luc J. C. van Loon

**Affiliations:** 1 TiFN, Wageningen, The Netherlands; 2 Department of Human Biology, NUTRIM School of Nutrition and Translational Research in Metabolism, Maastricht University Medical Centre+, P.O. Box 616 6200 MD, Maastricht, The Netherlands; 3 Division of Human Nutrition & Health, Department of Agrotechnology and Food Sciences, Wageningen University, Wageningen, The Netherlands

**Keywords:** Muscle protein synthesis, Plant-based proteins, Dairy, Protein blends, Fractional synthesis rate, Young healthy males

## Abstract

Plant-derived proteins are generally believed to possess lesser anabolic properties when compared with animal-derived proteins. This is, at least partly, attributed to the lower leucine content of most plant-derived proteins. Corn protein has a leucine content that is highest among most plant-derived proteins and it even exceeds the levels observed in animal-derived proteins such as whey protein. Therefore, this study aimed to compare muscle protein synthesis rates following the ingestion of 30 g corn protein and a 30 g blend of corn plus milk protein with 30 g milk protein. In a randomized, double blind, parallel-group design, 36 healthy young males (26 ± 4 y) received primed continuous L-[ring-^13^C_6_]-phenylalanine infusions and ingested 30 g corn protein (CORN), 30 g milk protein (MILK), or a 30 g proteinblend with 15 g corn plus 15 g milk protein (CORN + MILK). Blood and muscle biopsies were collected for 5 h following protein ingestion to assess post-prandial plasma amino acid profiles and myofibrillar protein synthesis rates. The results show that Ingestion of protein increased myofibrillar protein synthesis rates from basal post-absorptive values in all treatments(P < 0.001). Post-prandial myofibrillar protein synthesis rates did not differ between CORN vs MILK (0.053 ± 0.013 vs 0.053 ± 0.013%∙h^−1^, respectively; t-test P = 0.90), or between CORN + MILK vs MILK (0.052 ± 0.024 vs 0.053 ± 0.013%∙h^−1^, respectively; t-test P = 0.92). Ingestion of 30 g corn protein, 30 g milk protein, or a blend of 15 g corn plus 15 g milk protein robustly increases muscle protein synthesis rates in young males. The muscle protein synthetic response to the ingestion of 30 g corn-derived protein does not differ from the ingestion of an equivalent amount of milk protein in healthy, young males. Clinical Trial Registry number. NTR6548 (registration date: 27–06-2017) https://www.trialregister.nl/.

**Supplementary Information:**

The online version contains supplementary material available at 10.1007/s00726-023-03377-z.

## Introduction

Protein ingestion increases muscle protein synthesis rates (Gorissen et al. 2015; Groen et al. 2015). The increase in muscle protein synthesis rate is driven by the post-prandial rise in circulating plasma essential amino acid (EAA) concentrations (Volpi et al. 2003), with the increase in plasma leucine concentration being of particular relevance (Koopman et al. 2007, 2005; Rieu et al. 2006; Wall et al. 2013; Wilkinson et al. 2013). It has been hypothesized that the skeletal muscle anabolic properties of different types of protein are largely determined by their protein digestion and amino acid absorption kinetics and their specific amino acid profile (Boirie et al. 1997; Koopman et al. 2009; Tang and Phillips 2009). More specifically, it has been suggested that the ingestion of protein sources with lower EAA content and/or digestibility result in an attenuated anabolic response when compared with the ingestion of the same amount of protein derived from a higher quality protein source (Burd et al. 2012b; Pennings et al. 2011; Tang et al. 2009). In this regard, plant-based protein sources are suggested to possess lesser anabolic properties when compared to animal-based protein sources. In accordance, we have previously shown that the ingestion of 35 g wheat-derived protein, which is relatively low in leucine, lysine and methionine, fails to stimulate muscle protein synthesis rates when compared with the ingestion of an equivalent amount of casein protein in older adults (Gorissen et al. 2016). Soy protein, on the other hand, contains sufficient amounts of all EAAs according to the WHO/FAO/UNU amino acid requirements (Who/Fao/Unu Expert Consultation 2007), and has been shown to result in lower (Tang et al. 2009; Wilkinson et al. 2007; Yang et al. 2012) or similar (Churchward–Venne et al. 2019; Phillips et al. 2009) post-prandial muscle protein synthesis rates when compared to the ingestion of milk or whey protein in both young and older adults. However, to define the extent to which plant-based proteins can stimulate post-prandial muscle protein synthesis rates, more plant-derived proteins, besides wheat and soy, should be investigated.

Corn is a widely consumed cereal grain, providing mainly carbohydrates. However, it is also a plant based protein source. While plant-derived proteins generally contain less leucine when compared to animal-derived proteins, we previously showed that the leucine content of corn derived protein concentrates is surprisingly high with leucine levels as high as ~ 13% of the total protein content (Gorissen et al. 2018). In comparison, this is higher than the leucine content of whey protein (~ 11%) which is regarded as one of the most anabolic animal-derived protein sources (Gorissen et al. 2018). So far, no studies have assessed the muscle protein synthetic response to the ingestion of corn derived protein in vivo in humans.

Although leucine is regarded as the most important EAA to stimulate muscle protein synthesis (Devries et al. 2018; Dickinson et al. 2014; Wall et al. 2013), the presence of other EAAs also plays an important role in providing the required building blocks to support the post-prandial rise in muscle protein synthesis rates. Insufficient post-prandial availability of one (or more) of the EAA may attenuate the post-prandial rise in muscle protein synthesis rates and, as such, compromise the post-prandial anabolic response. It has been suggested that plant-derived proteins are less likely to increase muscle protein synthesis due to deficiencies in specific amino acids such as lysine and methionine (Van Vliet et al. 2015). In this regard, corn protein has a very high leucine content, but a very low lysine content. However, combining various protein sources in a blend may represent an effective strategy to provide a more balanced amino acid profile (Reidy et al. 2013; Young and Pellett 1994) and as such prevent any amino acid deficiencies. Since more than half of the worldwide protein consumption originates from plants (Faostat 2013), blends of both plant and animal-based protein may represent an effective and practical strategy to improve the overall quality, anabolic properties, and sustainability of the ingested protein.

We hypothesize that the ingestion of 30 g milk protein results in higher post-prandial muscle protein synthesis rates when compared with the ingestion of the same amount of corn protein. However, when corn and milk protein are combined in a 1/1 ratio, we expect these differences to disappear. To test these hypotheses, we selected 36 healthy young males to partake in a study in which we compared the impact of ingesting 30 g milk protein with the ingestion of 30 g corn protein or a protein blend of 15 g corn plus 15 g milk protein on post-prandial muscle protein synthesis rates in vivo in humans.

## Subjects and methods

### Participants

Thirty-six healthy males (26 ± 4 y; 1.78 ± 0.06 m; 72.5 ± 7.5 kg) volunteered to participate in this parallel group, double blind, randomized controlled trial (participants’ characteristics are presented in Table 1). Participants were recreationally active and generally performed between 2 and 4 exercise sessions per week in various sports (e.g. soccer, basketball, weight lifting, running, cycling, etc.), but were not involved in any structured progressive exercise training regimen. This study was part of a larger trial registered at the Netherlands Trial Register (NTR6548, https://www.trialregister.nl/trial/6364), and was conducted between June 2017 and April 2019 at Maastricht University in Maastricht, The Netherlands (See Online Resource 1 for the CONSORT (Consolidated Standards of Reporting Trials) flow diagram) (Pinckaers et al. 2021). All participants were informed about the purpose of the study, the experimental procedures, and possible risks before providing informed written consent to participate. The procedures followed were in accordance with the ethical standards of the medical ethics committee of Maastricht University Medical Centre + (METC 173001), and in accordance with the Helsinki Declaration of 1975 as revised in October 2013. The study was independently monitored and audited by the Clinical Trial Centre Maastricht.

**Table 1:** Participants’ characteristics

	MILK	CORN + MILK	CORN
Age (y)	26 ± 4	26 ± 5	27 ± 3
Height (m)	1.76 ± 0.06	1.76 ± 0.05	1.81 ± 0.07
Weight (kg)	71.5 ± 9.0	72.4 ± 6.9	73.7 ± 7.0
BMI (kg∙m^−2^)	23.0 ± 2.1	23.4 ± 2.0	22.4 ± 1.7
Systolic blood pressure (mmHg)	119 ± 6	114 ± 7	124 ± 9
Diastolic Blood Pressure (mmHg)	71 ± 9	66 ± 7	69 ± 9
Resting heart rate (bpm)	64 ± 10	65 ± 12	61 ± 7
Lean mass (kg)	53.2 ± 7.9	53.5 ± 4.7	56.9 ± 6.0
Body fat (%)	23.1 ± 3.2	22.5 ± 4.8	20.2 ± 4.7

Values represent mean ± standard deviation. n = 12 per nutritional intervention group. MILK: 30 g milk protein, CORN+MILK: 15g corn protein plus 15 g milk protein, CORN: 30 g of corn protein. Independent samples T-test for MILK vs CORN and MILK vs CORN+MILK all P>0.05

### Preliminary testing

Participants aged 18–35 y, with BMI > 18.5 and < 27.5 kg∙m^−2^ underwent an initial screening session to assess eligibility. Height, weight, blood pressure and body composition (by dual-energy X-ray absorptiometry; Discovery A, Hologic; (National Health and Nutrition Examination Survey—Body composition analysis (NHANES BCA) enabled) were determined. Participants were deemed healthy based on their responses to a medical questionnaire. The screening sessions and experimental trials were separated by at least 3 days.

### Study design

Participants were randomly assigned to ingest a 400 mL beverage containing either 30 g corn protein isolate (CORN), 30 g milk protein concentrate (MILK), or 15 g corn protein isolate plus 15 g milk protein concentrate (CORN + MILK). After beverage ingestion, the bottle was rinsed with 150 mL of water, which was also ingested by the participants. Milk protein concentrate (Refit MPC80) was obtained from FrieslandCampina (Wageningen, the Netherlands) and corn protein isolate was supplied by Cargill (Minneapolis, MN, USA). Participants were allocated to a treatment according to a block randomization list performed using a computerized randomizer (http://www.randomization.com/). An independent researcher was responsible for random assignment (*n* = 12 per group) and preparation of the study treatment beverages, which were sequentially numbered according to subject number. The beverages were prepared in non-transparent protein-shakers.

### Diet and physical activity

Participants refrained from sports and strenuous physical activities (e.g. lifting heavy weights), and alcohol consumption for 3 days prior to the experimental trial. In addition, all participants were instructed to complete a food and activity record for 3 days prior to the experimental trial. (See Online Resource 4 for an overview of participants’ habitual food intake in the 3 days prior to the experimental trial). The evening before the trial, all participants consumed a standardized meal containing 2.8 MJ, with 65% energy provided as carbohydrate, 20% as fat, and 15% as protein, before 10:00 PM after which they remained fasted.

### Experimental protocol

**Fig. 1:**
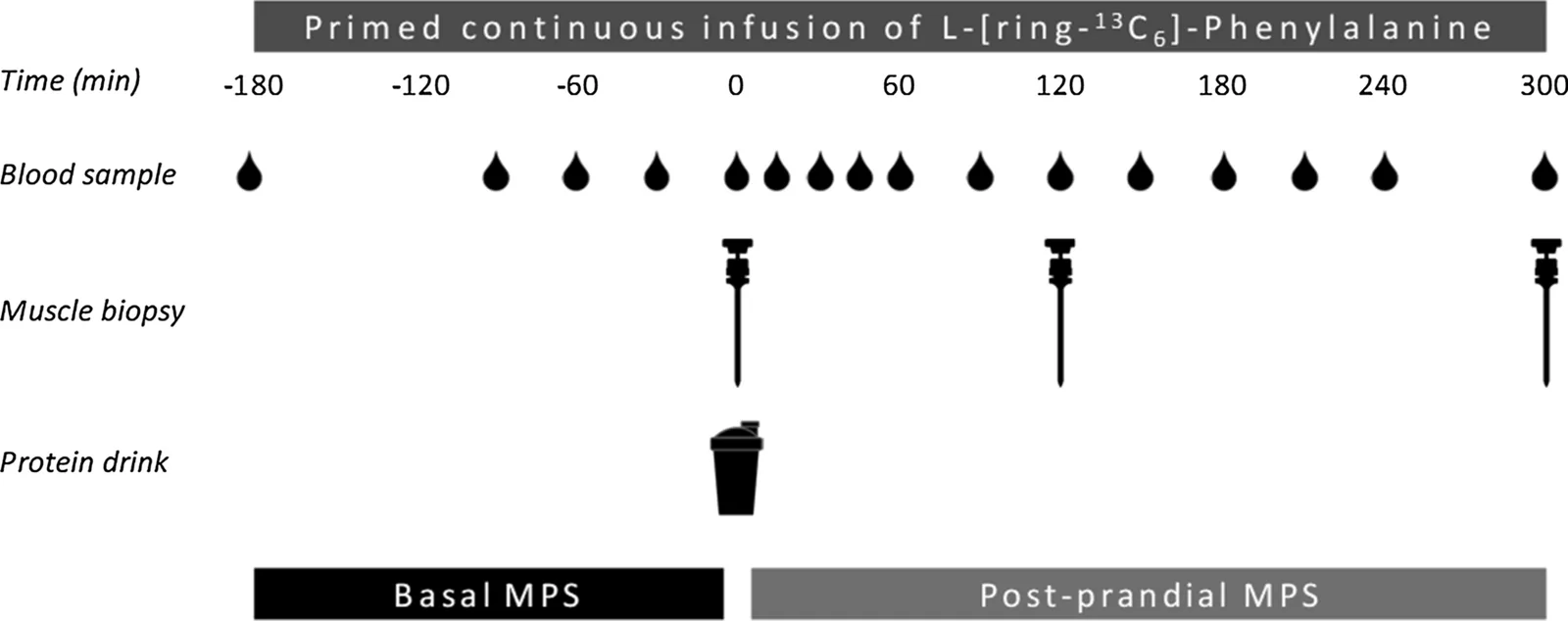
Schematic representation of the experimental design

At ~ 7:30 AM, participants arrived at the laboratory in an overnight post-absorptive state. A cannula was inserted into an antecubital vein for stable isotope amino acid infusion. A second cannula was inserted into a dorsal hand vein on the contralateral arm for arterialized blood sampling. To obtain arterialized blood samples, the hand was placed in a hot box (60 °C) for 10 min prior to blood sample collection. For a schematic representation of the experimental protocol, see Fig. 1.

After taking a baseline blood sample (*t* = − 180 min), the plasma phenylalanine pool was primed with a single dose of L-[ring-^13^C_6_]-phenylalanine (2.25 µmol∙kg^−1^). Thereafter, a continuous intravenous infusion of L-[ring-^13^C_6_]-phenylalanine (0.05 µmol∙kg^−1^∙min^−1^) was initiated (*t* = − 180 min) using a calibrated IVAC 598 pump (San Diego, CA, USA). Subsequently, arterialized blood samples were collected at *t* = − 90, − 60 and − 30 min. At *t* = 0 min an arterialized blood sample was obtained as well as a muscle biopsy from the *m. vastus lateralis*. Immediately following the muscle biopsy, participants ingested a 400 mL beverage corresponding to their randomized treatment allocation i.e.: CORN (*n* = 12), MILK (*n* = 12), or CORN + MILK (*n* = 12). To minimize dilution of the steady-state plasma L-[ring-^13^C_6_]-phenylalanine precursor pool, the phenylalanine content of the protein drink was enriched with 3.85% L-[ring-^13^C_6_]-phenylalanine. Arterialized blood samples were then collected at *t* = 15, 30, 45, 60, 90, 120, 150, 180, 210, 240, and 300 min after protein ingestion in the post-prandial period. Blood samples were collected into EDTA-containing tubes and centrifuged at 1200* g* for 10 min at 4 °C. Aliquots of plasma were frozen in liquid nitrogen and stored at − 80 °C. A second and third muscle biopsy from the *m. vastus lateralis* were collected at *t* = 120 and *t* = 300 min to determine post-prandial skeletal muscle protein synthesis rates over the 0–120, 120–300, and 0–300 min post-prandial periods. Muscle biopsy collection was alternated between legs and obtained with the use of a 5 mm Bergström needle (Bergstrom 1975), custom-adapted for manual suction. Samples were obtained from separate incisions from the middle region of the *m. vastus lateralis*, ~ 15 cm above the patella and ~ 3 cm below entry through the fascia. Local anesthetic (1% Xylocaine with adrenaline 1:100,000) was applied to numb the skin and fascia. Muscle samples were freed from any visible non-muscle material, immediately frozen in liquid nitrogen, and stored at − 80 °C until further processing. When the experimental protocol was complete, cannulae were removed and participants were provided with food and monitored for ~ 30 min before leaving the laboratory.

### Protein powder analysis

Batch-specific nitrogen contents of both milk protein concentrate and corn protein isolate were provided by the manufacturer. The protein content of milk protein powder was determined as nitrogen content × 6.38 and the protein content of corn protein powder was determined as nitrogen content × 6.25 (Jones DB, 1941; Mariotti et al. 2008). Amino acid contents of the protein powders were determined by acid hydrolysis in triplicate, as previously described (Pinckaers et al. 2021). Tryptophan contents were obtained by alkaline hydrolysis, as performed by La Cour et al. (La Cour et al. 2019). In short, ~ 10 mg of the protein samples was weighed in glass screw cap vials. Subsequently, a 3 mL solution with 95 mM ascorbic acid and 4 M sodium hydroxide was added to the sample and heated to 110 °C for 16 h. Subsequent analysis of the free amino acids was performed using ultra-performance liquid chromatography-mass spectrometry (UPLC-MS; ACQUITY UPLC H-Class with QDa; Waters, Saint-Quentin, France), as previously described (Pinckaers et al. 2021). The amino acid composition of the protein powders and protein blend are presented in Table 2. It is worthwhile to note that the use of a single protein hydrolysis step is suboptimal for a quantitative assessment of all amino acids (Darragh and Moughan 2005), resulting in the underestimation of the sulphur containing amino acid contents and the inability to assess tryptophan concentrations.

**Table 2:** Amino acid composition of the provided proteins

	MILK	CORN + MILK^1^	CORN
Alanine	0.9	1.6	2.3
Arginine	0.8	0.7	0.6
Aspartic acid + Asparagine	1.8	1.6	1.4
Cystine	0.1	0.1	0.2
Glutamic acid + Glutamine	5.1	5.3	5.5
Glycine	0.5	0.6	0.7
Histidine	0.6	0.5	0.4
Isoleucine	0.9	0.7	0.6
Leucine	2.4	3.3	4.1
Lysine	2.0	1.2	0.3
Methionine	0.7	0.7	0.6
Phenylalanine	1.2	1.4	1.5
Proline	2.9	2.8	2.7
Serine	1.2	1.3	1.4
Threonine	0.9	0.9	0.8
Tryptophan	0.4	0.3	0.1
Tyrosine	0.6	0.6	0.6
Valine	1.1	0.9	0.7
TAA	24.2	24.4	24.6
EAA	10.2	9.7	9.2
BCAA	4.4	4.9	5.4
Nitrogen content (%)	13.4	13.8	14.4
Protein content (%)	85.5^3^	87.8	90.0^2^

Values for amino acid contents are in grams per 30 g protein

*MILK* 30 g milk protein, *CORN + MILK* 15 g corn protein plus 15 g milk protein, CORN 30 g of corn protein, *BCAA* branched chain amino acids, *EAA* essential amino acids, *TAA* total amino acids

^1^Values are obtained by averaging the measured values for corn and milk protein

^2^Protein as nitrogen content * 6.38

^3^Protein as nitrogen content * 6.25

### Plasma analysis

Plasma glucose and insulin concentrations were analyzed using commercially available kits (ref. no. A11A01667, Glucose HK CP, ABX Diagnostics, Montpellier, France; and ref. no. HI-14 K, Millipore, St. Louis, MO, respectively). Plasma amino acid concentrations were determined by UPLC-MS, as previously described (Pinckaers et al. 2021).

Plasma L-[ring-^13^C_6_]-phenylalanine enrichments were determined by gas chromatography-mass spectrometry (GC–MS; Agilent 7890A GC/5975C MSD; Agilent Technologies), as previously described (Pinckaers et al. 2021). In short, the free amino acids from deproteinized plasma samples were purified using cation exchange resin columns (AG 50W-X8, mesh size: 100–200, ionic form: hydrogen (Bio-Rad Laboratories, Hercules, CA, USA)), and subsequently converted to their tert-butyl dimethylsilyl (TBDMS) derivative before analysis by GC–MS.

Basal muscle protein synthesis rates were assessed to confirm that protein ingestion increases muscle protein synthesis rates. The single biopsy approach was applied to assess post-absorptive muscle protein synthesis rates without the need to collect and additional muscle biopsy (Burd et al. 2012a). In short, plasma protein obtained prior to tracer infusion (*t* = − 180 min) was used to determine background L-[ring-^13^C_6_]-phenylalanine enrichments. For this purpose, the plasma sample was precipitated by adding perchloric acid. Subsequently, similarly as for the myofibrillar protein fraction, the denaturized plasma protein pellet was hydrolyzed, passed over a cation exchange resin column (AG 50W-X8, mesh size: 100–200, ionic form: hydrogen (Bio-Rad Laboratories, Hercules, CA, USA)), and the resulting amino acid samples were derivatized to their N(O,S)-ethoxycarbonyl-ethylesters before being measured by gas chromatography-combustion-isotope ratio mass spectrometry (GC-IRMS; Mat 253, Thermo Scientific, Bremen, Germany) using a DB5MS (30 m) column (Agilent technologies, Santa Clara, Ca, USA), as previously described (Pinckaers et al. 2021).

### Muscle analysis

Muscle analysis for the determination of muscle protein bound L-[ring-^13^C_6_]-phenylalanine enrichments has previously been explained in detail (Pinckaers et al. 2021). In short, a piece of wet muscle (~ 50–70 mg) was homogenized and a myofibrillar protein-enriched fraction was obtained by removal of the collagen-enriched fraction. Subsequently, the amino acids were liberated from the myofibrillar protein-enriched fraction by adding 2 mL of 6 M HCl and heating to 110 °C for 16 h. The amino acids from the resulting dried myofibrillar protein-enriched fractions were liberated by adding 2 mL of 6 M HCl and heating to 110 °C for 16 h, passed over a cation exchange resin column (AG 50W-X8, mesh size: 100–200, ionic form: hydrogen (Bio-Rad Laboratories, Hercules, CA, USA)), and derivatized to their N(O,S)-ethoxycarbonyl-ethylesters. The ratio of ^13^C/^12^C of myofibrillar protein-bound phenylalanine was determined using GC-IRMS. Muscle intra-cellular enrichments were determined from a separate piece of muscle, as described elsewhere (Pinckaers et al. 2021).

### Calculations

Fractional myofibrillar protein synthesis rates (%∙h^−1^) were calculated by the standard precursor-product equation (Schierbeek H 2017):

$$FSR=\left(\frac{\left({E}_{b2}-{E}_{b1}\right)}{\left({E}_{precursor}\bullet t\right)}\right)\bullet 100,$$

where E_b_ is the increment in myofibrillar protein-bound L-[ring-^13^C_6_]-phenylalanine enrichment (mole % excess, MPE) during the tracer incorporation period, and t is the tracer incorporation time in h. Weighted mean plasma L-[ring-^13^C_6_]-phenylalanine enrichments were calculated by taking the measured enrichment between consecutive time points and correcting for the time between these sampling time points (E_precursor_). For calculation of post-prandial FSR, skeletal muscle biopsy samples at *t* = 0, 120 and 300 min were used. For the calculation of basal FSR, E_b2_ represented the protein-bound L-[ring-^13^C_6_]-phenylalanine enrichments in muscle at *t* = 0 min, and E_b1_ represented the protein bound L-[ring-^13^C_6_]-phenylalanine enrichments in plasma protein at *t* = − 180 min.

Net incremental area under curve (iAUC) was determined for plasma amino acid concentrations during the 5 h post-prandial period following protein ingestion. The iAUC was calculated using the trapezoid rule, with plasma concentrations before beverage ingestion (*t* = 0 min) serving as baseline.

### Outcome measures

Myofibrillar FSR over the entire (i.e. 0–300 min) post-prandial period, comparing MILK *vs* CORN and MILK *vs* CORN + MILK was defined as the primary outcome measure. Secondary outcome measures were myofibrillar FSR in the early (i.e. 0–120 min) and late (i.e. 120–300 min) post-prandial period, plasma glucose, insulin, and amino acid concentrations and plasma amino acid iAUC, comparing MILK *vs* CORN and MILK *vs* CORN + MILK. Plasma glucose, insulin, and amino acid peak concentrations and time to peak were tertiary outcomes, comparing MILK *vs* CORN and MILK *vs* CORN + MILK.

### Statistical analysis

A power calculation was performed with differences in postprandial myofibrillar FSRs between 2 treatments as primary outcome measure. Based on previous work in this area, a sample size of 12 participants per treatment, including a 10% dropout rate was calculated using a power of 80%, a significance level of 0.05, a difference in FSR of 0.008%∙h^−1^ (or ~ 20% when expressed as relative difference, e.g. 0.040 vs 0.048%∙h^−1^) (Witard et al. 2014), and a within-group standard deviation of 0.0065%∙h^−1^ (or ~ 16%) (Moore et al. 2009; Trommelen et al. 2016). Participants’ characteristics were analyzed by independent samples *t*-test for MILK vs CORN and MILK *vs* CORN + MILK. Plasma glucose, insulin, and amino acid concentrations and amino acid enrichments over time were compared between groups using a two-way (*Time x treatment*) repeated measures ANOVA for MILK *vs* CORN and MILK *vs* CORN + MILK, with time as within-subjects factor, and treatment as between-subjects factor. In case a significant *time x treatment* interaction was observed, independent samples *t*-tests were performed to determine significant differences between treatments for each time point. Plasma glucose, insulin, and amino acid concentrations, expressed as peak values, time to peak and iAUC, were analyzed by independent samples *t*-test for MILK *vs* CORN and MILK *vs* CORN + MILK. Basal post-absorptive, and post-prandial myofibrillar protein synthesis rates during the early (0–120 min) and entire (0–300 min) post-prandial period were analyzed by independent samples *t*-test for MILK *vs* CORN and MILK *vs* CORN + MILK. Similarly, within group post-absorptive *vs* post-prandial myofibrillar protein synthesis rates were analyzed by independent samples *t*-test. Statistical analyses were performed with a software package (IBM SPSS statistics for Windows, version 26.0, IBM Corp., Armonk, NY, USA). Means were considered to be significantly different for *P* values < 0.05. Data are expressed as means ± SD.

## Results

### Plasma glucose and insulin concentrations

**Fig. 2:**
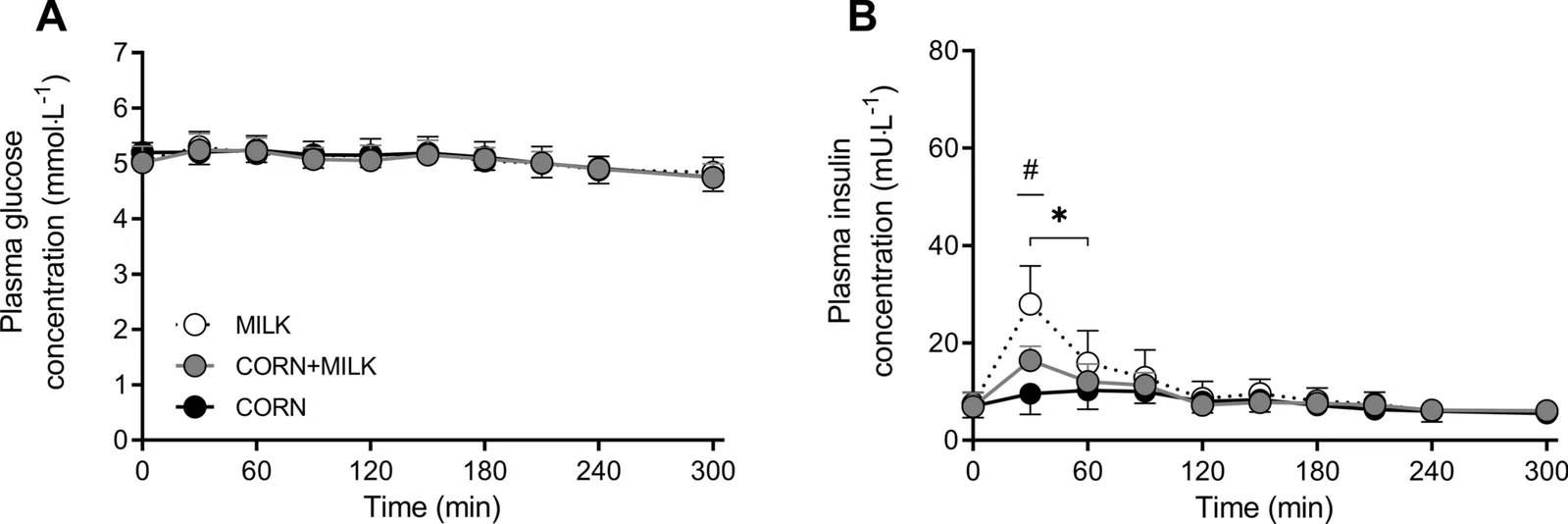
Post-prandial plasma glucose (Panel A) and insulin (Panel B) concentrations during the 300 min period following the ingestion of MILK *vs* CORN and MILK *vs* CORN + MILK in healthy young males (*n* = 12 per group). Time 0 min represents time of beverage intake. MILK: 30 g milk protein, CORN: 30 g corn protein, CORN + MILK: 15 g corn protein + 15 g milk protein. Values represent means ± standard deviation; Repeated measures ANOVA with time as within-subject variable and interventional drink (treatment) as between-subject variable. *Time x treatment*: Panel A: MILK *vs* CORN *P* = 0.02, MILK *vs* CORN + MILK *P* = 0.33; Panel B: MILK *vs* CORN *P* < 0.001, MILK *vs* CORN + MILK *P* < 0.001. * Significantly different for MILK *vs* CORN (*P* < 0.05), ^#^ significantly different for MILK *vs* CORN + MILK (*P* < 0.05)

Plasma glucose concentrations were maintained following protein ingestion (Fig. 2A). Although a significant *Time x treatment* interaction was observed (*P* = 0.02), no differences at individual time points were observed in glucose concentrations between MILK *vs* CORN (*P* > 0.05). Plasma glucose concentrations did not change for MILK *vs* CORN + MILK (*Time x treatment*: *P* = 0.33; *Time: P* < 0.001). Plasma insulin concentrations increased following protein ingestion, with MILK reaching higher peak concentrations when compared to CORN (28 ± 8 *vs* 11 ± 4 mU∙L^−1^, respectively, *P* < 0.001) and compared to CORN + MILK (28 ± 8 *vs* 17 ± 3 mU∙L^−1^, respectively, *P* < 0.001; Fig. 2B). The insulin iAUC was greater for MILK *vs* CORN (1058 ± 331 *vs* 232 ± 335 mU^−1^ respectively, *P* < 0.001) as well as for MILK *vs* CORN + MILK (1058 ± 331 *vs* 584 ± 575, respectively, *P* = 0.03).

### Plasma AA concentrations

**Fig. 3:**
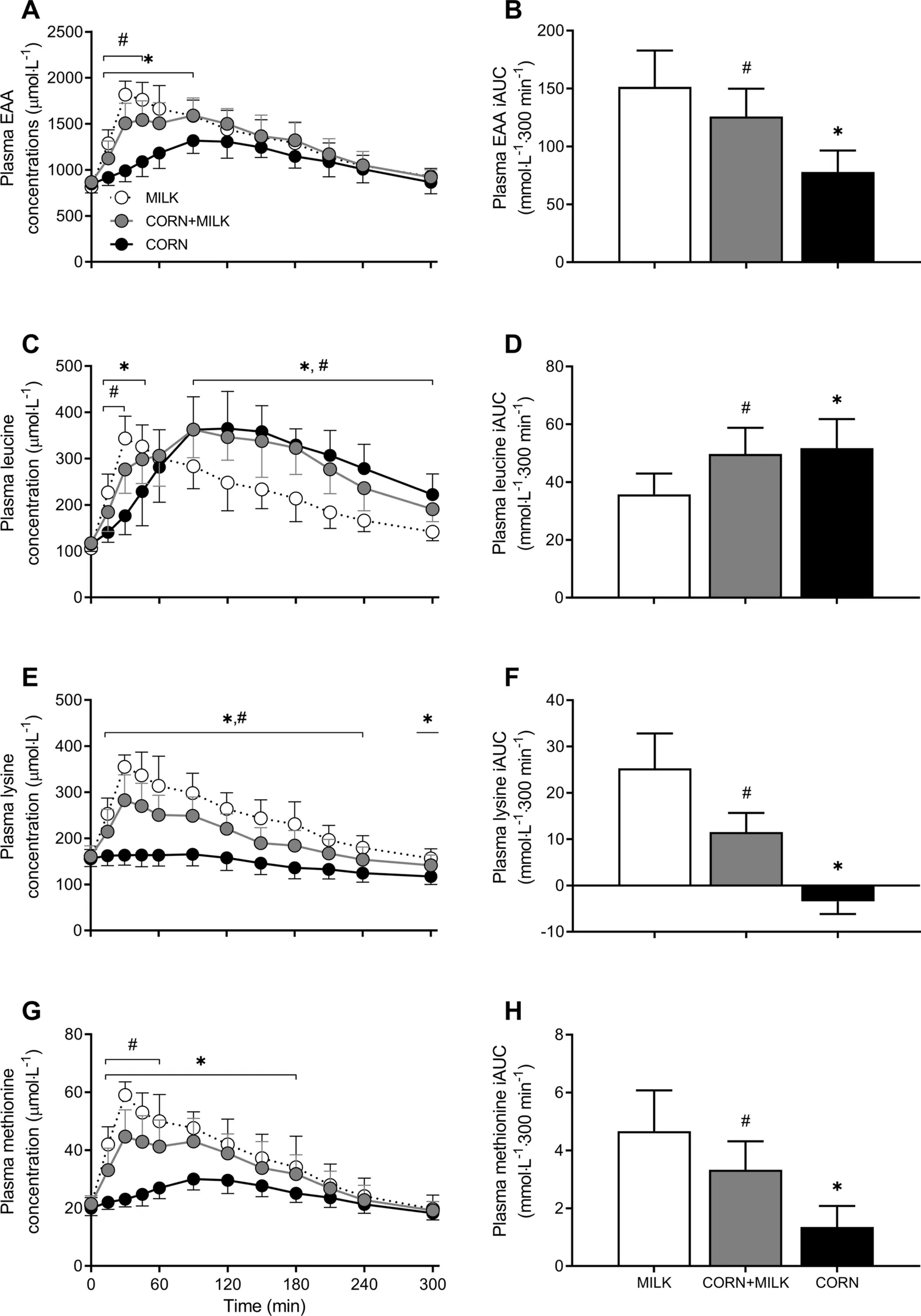
Post-prandial plasma essential amino acid (EAA, Panel A), leucine (Panel C), lysine (Panel E), and methionine (Panel G) concentrations during the 300 min period following the ingestion of MILK *vs* CORN and MILK *vs* CORN + MILK in healthy, young males (*n* = 12 per group). Time 0 min represents time of beverage intake. Panels B, D, F and H represent the 0–5 h incremental area under curve (iAUC) following protein ingestion. MILK: 30 g milk protein, CORN: 30 g corn protein, CORN + MILK: 15 g corn protein + 15 g milk protein. Values represent means ± standard deviation; * significantly different for MILK *vs* CORN (*P* < 0.05), ^#^ significantly different for MILK *vs* CORN + MILK (*P* < 0.05). Repeated measures ANOVA with time as within-subject variable and interventional drink (treatment) as between-subject variable. *Time x treatment*: Panel A: MILK *vs* CORN *P* < 0.001, MILK *vs* CORN + MILK *P* < 0.01, Panel C: MILK *vs* CORN* P* < 0.001, MILK *vs* CORN + MILK *P* < 0.001, Panel E: MILK *vs* CORN* P* < 0.001, MILK *vs* CORN + MILK *P* < 0.01, Panel G: MILK *vs* CORN* P* < 0.001, MILK *vs* CORN + MILK *P* < 0.01

Plasma EAA concentrations increased following protein ingestion for all treatments (Fig. 3A). This increase was greater for both MILK *vs* CORN and MILK vs CORN + MILK (*Time x treatment*: *P* < 0.001 and *P* < 0.01, respectively). MILK ingestion resulted in higher peak EAA concentrations *vs* CORN (1871 ± 124 *vs* 1355 ± 152 µmol∙L^−1^; *P* < 0.001) and *vs* CORN + MILK (1871 ± 124 *vs* 1684 ± 176 µmol∙L^−1^; *P* < 0.001). These peak EAA concentrations were reached faster following MILK *vs* CORN (36 ± 10 *vs* 108 ± 27 min; *P* < 0.001), and following MILK *vs* CORN + MILK (36 ± 10 *vs* 91 ± 46 min; *P* < 0.001). The overall increase in plasma EAA concentrations over the entire 300 min post-prandial period, expressed as iAUC, was 94% greater for MILK *vs* CORN (151 ± 31 *vs* 78 ± 19 mmol∙300 min∙L^−1^; *P* < 0.001) and 20% greater for MILK *vs* CORN + MILK (151 ± 31 *vs* 126 ± 24 mmol∙300 min∙L^−1^; *P* = 0.04; Fig. 3B).

Plasma leucine concentrations increased over time for all treatments following protein ingestion (Fig. 3C). This increase differed significantly for both MILK *vs* CORN and MILK *vs* CORN + MILK (*Time x treatment*, both *P* < 0.001). Although peak plasma leucine concentrations did not differ between MILK *vs* CORN (353 ± 45 *vs* 390 ± 66 µmol∙L^−1^; *P* = 0.12) and MILK *vs* CORN + MILK (353 ± 45 *vs* 395 ± 62 µmol∙L^−1^; *P* = 0.07), MILK reached peak plasma leucine concentrations earlier when compared to CORN (46 ± 43 vs 130 ± 35 min, respectively, *P* < 0.001) and CORN + MILK (46 ± 43 *vs* 133 ± 45 min, respectively, *P* < 0.001). From 90 min onwards, plasma leucine concentrations were higher in both CORN *vs* MILK and CORN + MILK *vs* MILK. The overall increase in plasma leucine concentrations over the entire 300 min post-prandial period, expressed as iAUC, was 45% greater for CORN *vs* MILK (52 ± 10 *vs* 36 ± 7 mmol∙300 min∙L^−1^; *P* < 0.001), and 39% greater for CORN + MILK *vs* MILK (50 ± 9 *vs* 36 ± 7 mmol∙300 min∙L^−1^; *P* < 0.01; Fig. 3D).

Plasma lysine concentrations increased over time for MILK and CORN + MILK, but not for CORN (Fig. 3E). This increase was greater for MILK *vs* CORN (*Time x treatment*: *P* < 0.001), as well as for MILK *vs* CORN + MILK (*Time x treatment*: *P* < 0.01). MILK ingestion resulted in higher peak lysine concentrations *vs* CORN (370 ± 29 *vs* 174 ± 25 µmol∙L^−1^; *P* < 0.001) and *vs* CORN + MILK (370 ± 29 *vs* 289 ± 46 µmol∙L^−1^; *P* < 0.001). Time to reach these peak concentrations was shorter for MILK *vs* CORN (34 ± 7 *vs* 73 ± 36 min, respectively; *P* = 0.001), but did not differ for MILK *vs* CORN + MILK: 34 ± 7 *vs* 43 ± 19 min; *P* = 0.15). The overall increase in plasma lysine concentrations over the entire 300 min post-prandial period, expressed as iAUC, was much greater for MILK *vs* CORN (25 ± 8 *vs* -3 ± 3 mmol∙300 min∙L^−1^; *P* < 0.001), and 119% greater for MILK *vs* CORN + MILK (25 ± 8 vs 12 ± 4 mmol∙300 min∙L^−1^; *P* < 0.001; Fig. 3F).

Plasma methionine concentrations increased over time for all treatments following protein ingestion (Fig. 3G). This increase was greater for MILK *vs* CORN (*Time x treatment*: *P* < 0.001), as well as for MILK *vs* CORN + MILK (*Time x treatment*: *P* < 0.01). MILK ingestion resulted in higher peak methionine concentrations *vs* CORN (60 ± 5 *vs* 31 ± 4 µmol∙L^−1^; *P* < 0.001) and *vs* CORN + MILK (60 ± 5 *vs* 47 ± 8 µmol∙L^−1^; *P* < 0.001). These peak methionine concentrations were reached faster following MILK ingestion *vs* CORN (34 ± 9 *vs* 103 ± 24 min; *P* < 0.001), and *vs* CORN + MILK (34 ± 9 *vs* 53 ± 26 min; *P* = 0.03). The overall increase in plasma methionine concentrations over the entire 300 min post-prandial period, expressed as iAUC, was 5 times greater for MILK *vs* CORN (5 ± 1 *vs* 1 ± 1 mmol∙300 min∙L^−1^; *P* < 0.001), and 40% greater for MILK *vs* CORN + MILK (5 ± 1 *vs* 3 ± 1 mmol∙300 min∙L^−1^; *P* < 0.001; Fig. 3H).

In general, increases in plasma amino acid concentrations revealed significant differences over time between MILK and CORN for all measured amino acids (Online Resource 2, *Time x treatment* all *P* < 0.001). For MILK *vs* CORN + MILK, the increase in plasma amino acid concentrations was significantly different for all measured amino acids, except for cystine, glutamic acid, glycine, histidine, ornithine, threonine and valine (Online Resource 2). The increases in plasma amino acid concentrations over the entire 300 min post-prandial period (iAUC) were greater for glutamic acid, isoleucine, ornithine, proline, serine, threonine, tryptophan, tyrosine valine, BCAA, NEAA, and TAA for MILK *vs* CORN (*P* < 0.05). For MILK *vs* CORN + MILK, plasma iAUC were greater for isoleucine, threonine, tryptophan, and valine (*P* < 0.05, Online Resource 2).

### ***Plasma and muscle L-[ring-***^***13***^***C***_***6***_***]-phenylalanine enrichments***

**Fig. 4:**
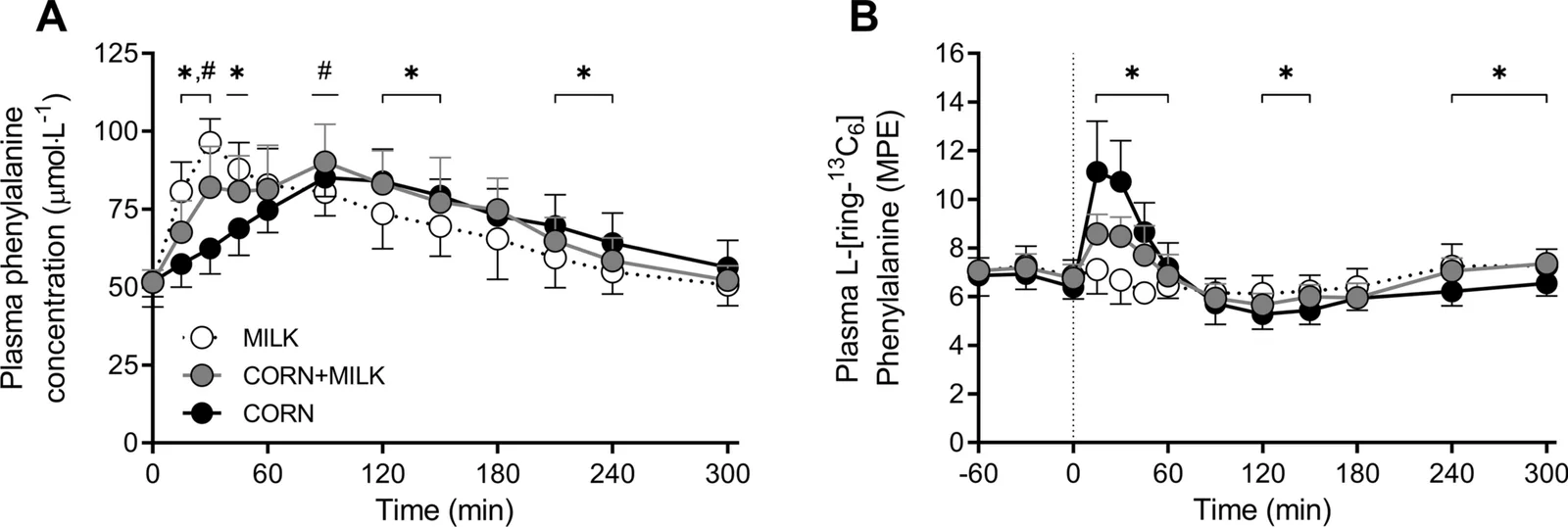
Post-prandial plasma phenylalanine concentrations (Panel A) and plasma 1-[^13^C_6_]-phenylalanine enrichments (Panel B) during the 300 min period following the ingestion of MILK *vs* CORN and MILK *vs* CORN + MILK in healthy, young males (*n* = 12 per group). Time 0 min represents time of beverage intake. MILK: 30 g milk protein, CORN: 30 g corn protein, CORN + MILK: 15 g corn protein + 15 g milk protein. Values represent means ± standard deviation; * significantly different for MILK *vs* CORN (*P* < 0.05), ^#^ significantly different for MILK *vs* CORN + MILK (*P* < 0.05). Repeated measures ANOVA with time as within-subject variable and interventional drink (treatment) as between-subject variable. *Time x treatment:* Panel A: MILK *vs* CORN *P* < 0.001, MILK *vs* CORN + MILK *P* < 0.001, Panel B: MILK *vs* CORN *P* < 0.001, MILK *vs* CORN + MILK *P* = 0.15

Plasma L-phenylalanine concentrations and L-[ring-^13^C_6_]-phenylalanine enrichments over time are presented in Fig. 4A and B, respectively. Plasma L-[ring-^13^C_6_]-phenylalanine enrichments over time were different between MILK *vs* CORN at *t* = 15, 30, 45, 60, 120, 150, 240, and 300 min following protein ingestion (*time x treatment: P* < 0.001), but not between MILK *vs* CORN + MILK (Fig. 4B; *time x treatment: P* = 0.15). Mean plasma L-[ring-^13^C_6_]-phenylalanine enrichments averaged 7.11 ± 0.65, 7.05 ± 0.43 and 6.78 ± 0.59 MPE during the basal post-absorptive period, and 6.64 ± 0.53, 6.72 ± 0.35, and 6.65 ± 0.33 MPE during the full 300 min post-prandial period for MILK, CORN + MILK, and CORN, respectively.

Myofibrillar protein-bound L-[ring-^13^C_6_]-phenylalanine enrichments increased following ingestion of MILK, CORN + MILK and CORN from 0.0032 ± 0.0032, 0.0033 ± 0.0032, and 0.0036 ± 0.0026 MPE at *t* = 0 min, to 0.0115 ± 0.0041, 0.0116 ± 0.0070, and 0.0124 ± 0.0045 MPE at *t* = 120 min, reaching 0.0214 ± 0.0049, 0.0214 ± 0.0108, and 0.0216 ± 0.0053 MPE, respectively, at 300 min after protein ingestion, with no differences observed between MILK *vs* CORN (all *P* > 0.65) and MILK *vs* CORN + MILK (all *P* > 0.93) at any time point.

### Muscle protein synthesis rates

**Fig. 5:**
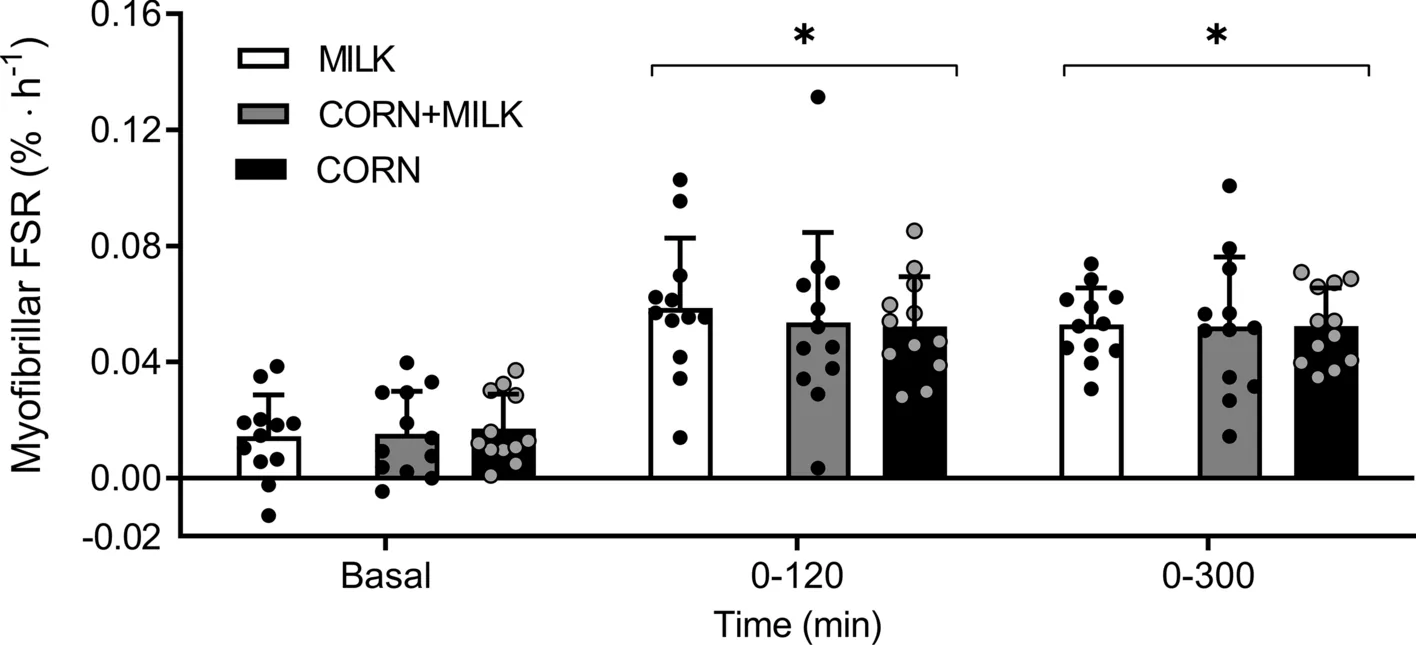
Myofibrillar fractional synthetic rate (FSR) at different time points following ingestion of CORN *vs* MILK and MILK *vs* CORN + MILK in healthy, young males (*n* = 12 per group). MILK: 30 g milk protein, CORN: 30 g corn protein, CORN + MILK: 15 g corn protein + 15 g milk protein. Bars represent means ± standard deviation, dots represent individual values. *significantly different from basal; *P* < 0.05. Independent samples *t*-test: MILK *vs* CORN *P* = 0.61, *P* = 0.46, and *P* = 0.90 for basal, 0–120, and 0–300 min, respectively. MILK *vs* CORN + MILK *P* = 0.88, *P* = 0.66, and *P* = 0.92 for basal, 0–120, and 0–300 min, respectively

Post-absorptive fractional myofibrillar protein synthesis rates averaged 0.014 ± 0.014, 0.015 ± 0.015 and 0.017 ± 0.012%∙h^−1^ in MILK, CORN + MILK, and CORN, with no differences between MILK *vs* CORN (*P* = 0.61) and MILK *vs* CORN + MILK (*P* = 0.88). Protein ingestion increased myofibrillar protein synthesis rates to 0.059 ± 0.024, 0.054 ± 0.031 and 0.052 ± 0.017%∙h^−1^ during the early post-prandial period (0–120 min) and to 0.049 ± 0.017, 0.051 ± 0.032, 0.052 ± 0.021%∙h^−1^ during the late post-prandial period (120–300 min). Post-prandial muscle protein FSR averaged 0.053 ± 0.013, 0.052 ± 0.024 and 0.052 ± 0.013%∙h^−1^ assessed over the entire 300 min post-prandial period after protein ingestion (Fig. 5). Post-prandial myofibrillar protein synthesis rates did not differ between MILK *vs* CORN, for the early (0–120 min; *P* = 0.46), late (120–300 min; *P* = 0.73), and entire (0–300 min; *P* = 0.90) post-prandial period. Similarly, post-prandial myofibrillar protein synthesis rates did not differ between MILK *vs* CORN + MILK, for the early (0–120 min; *P* = 0.66), late (120–300 min; *P* = 0.87), and entire (0–300 min; *P* = 0.92) post-prandial period (Fig. 5). Myofibrillar protein synthesis rates determined with the intra-cellular L-[ring-^13^C_6_]-phenylalanine enrichments used as precursor pool resulted in similar findings with no differences in FSR values between Milk *vs* CORN and MILK *vs* CORN + MILK at any time point (Online Resource 3).

## Discussion

The present study shows that ingestion of 30 g corn protein or a blend of corn plus milk protein blend robustly increases muscle protein synthesis rates in healthy, young males. Despite the observation of faster and greater post-prandial plasma EAA availability following milk when compared to corn or corn plus milk protein blend ingestion, post-prandial myofibrillar protein synthesis rates did not differ between treatments.

Corn is a plant-based protein source with an exceptionally high leucine content of ~ 13% of total protein content, when compared to ~ 8% in milk protein. As leucine availability represents a key factor for stimulating post-prandial muscle protein synthesis (Koopman et al. 2005; Rieu et al. 2006; Wall et al. 2013; Wilkinson et al. 2013), corn-derived protein may represent a plant derived protein that can robustly stimulate skeletal muscle protein synthesis rates. However, although corn-derived protein holds a very high leucine content, lysine content (1.5%) falls well below the WHO/FAO/UNU requirements of 4.5% (Gorissen et al. 2018; Who/Fao/Unu Expert Consultation 2007). The insufficient provision of one (or more) EAA could be restrictive and, as such, attenuate the post-prandial rise in muscle protein synthesis rates when compared to higher quality animal derived proteins (Gorissen et al. 2018; Van Vliet et al. 2015). Whether the relative high leucine content may be offset by the lysine deficiency in corn derived protein remains speculative. In the present study, we confirmed that the leucine content was substantially higher (4.1 *vs* 2.4 g, respectively) and the lysine content was considerably lower (0.3 *vs* 2.0 g, respectively) in the corn derived protein compared with the milk derived protein (Table 2). Ingestion of the proteins robustly increased circulating amino acid concentrations (Fig. 3). Although the increase in plasma leucine concentrations was more rapid following ingestion of milk when compared with the ingestion of corn-derived protein, the latter showed a more sustained increase resulting in an overall 45% greater plasma leucine availability over the entire 5 h post-prandial period (Fig. 3D). In line with the amino acid composition of the proteins, plasma lysine concentrations increased significantly following milk protein ingestion, whereas no increase above post-absorptive values was observed following ingestion of the corn derived protein. In accordance, post-prandial plasma lysine availability was significantly greater following milk when compared with corn protein ingestion, for which no increase in lysine concentration was detected. These results tend to be in line with previous reports on post-prandial amino acid responses following wheat protein ingestion, which is also particular low in lysine content, showing no significant changes in post-prandial plasma lysine availability following ingestion of, respectively, 30 and 35 g of wheat protein hydrolysate (Gorissen et al. 2016; Pinckaers et al. 2021). Though post-prandial plasma amino acid responses seem to follow differences in amino acid profiles of the ingested proteins, it is evident that these differences are certainly not proportional. This discrepancy is likely attributed to various differences in protein digestion, amino acid absorption, and/or amino acid retention in splanchnic tissues.

Previous work suggests that post-prandial plasma amino acid availability may be predictive for the anabolic response following protein ingestion (Church et al. 2020; Tang et al. 2009). In the present study, we observed a strong increase in muscle protein synthesis rates above basal post-absorptive rates following the ingestion of corn-derived protein. A response that did not differ from the response observed after ingesting an equivalent amount of milk protein (Fig. 5). Clearly, the provided corn-derived protein is capable of robustly stimulating muscle protein synthesis rates in vivo in humans. We can only speculate whether this is attributed to high(er) leucine content, as we recently also observed no differences in muscle protein synthesis rates following the ingestion of similar amounts of wheat (lower leucine content, (Pinckaers et al. 2021)) and potato (equal leucine content (Pinckaers et al. 2022))-derived protein when compared to an equivalent amount of dairy protein. Our data do indicate that the low lysine content provided in corn protein is not restrictive for the acute in vivo skeletal muscle anabolic response when ingesting an ample amount of protein. This seems also in line with our previous findings on the post-prandial response to wheat ingestion in young healthy adults, where we failed to detect any differences in muscle protein synthesis rates following ingestion of a sufficient amount (e.g. 30 g) of wheat when compared with milk-derived protein, despite its low lysine and methionine contents (Pinckaers et al. 2021). These unexpected findings may be attributed to the selection of healthy young volunteers in the present study, in contrast to the older individuals selected in the previous studies (Gorissen et al. 2016; Yang et al. 2012) who may have been suffering from some level of anabolic resistance. Alternatively, previous studies have provided ~ 20 g protein to evaluate differences in post-prandial muscle protein synthesis rates (Tang et al. 2009; Wilkinson et al. 2007), which is in line with the estimated ~ 0.25 g∙kg^−1^ body mass animal-derived protein needed to maximize post-prandial muscle protein synthesis. The optimal amount of plant-derived protein to be ingested for stimulating muscle protein synthesis remains to be determined. However, the 30 g protein dose (which represented ~ 0.40 g∙kg^−1^ body mass), provided in the current study, may represent a more than adequate amount to maximize muscle protein synthesis rates, regardless the animal or plant derived origin of the protein. Furthermore, in this acute setting, the high leucine content of corn-derived protein (Gorissen et al. 2018) may have been the main driver of the post-prandial muscle protein synthetic response. Obviously, the lower plasma lysine availability following ingestion of corn-derived when compared to milk-derived protein ingestion, did not restrict the acute post-prandial rise in muscle protein synthesis rate.

We anticipated that the low lysine content in corn derived protein would compromise the anabolic response following protein ingestion and, as such, we added a third treatment group with a blend of 50% corn derived and 50% milk protein. While for the comparison of milk *vs* corn protein, the protein lysine content amounted ~ 6.5 *vs* ~ 1%, respectively, for the milk *vs* corn plus milk protein comparison, the lysine content was ~ 6.5 *vs* ~ 4%, respectively. This resulted in the lysine content of the protein blend to approach the lysine requirements for adults of 4.5% as indicated by the WHO/FAO/UNU (Who/Fao/Unu Expert Consultation 2007). In line with the comparison with corn protein only, no differences were observed in post-prandial muscle protein synthesis rates following the ingestion of milk and the corn plus milk protein blend. The finding that both the corn protein and corn plus milk protein blend did not differ in their capacity to stimulate muscle protein synthesis rates when compared with milk protein, provides additional evidence that lysine deficiency in a plant-derived protein does not seem to compromise the acute post-prandial muscle protein synthetic response when compared to the ingestion of a higher quality, animal-derived protein, under conditions where a sufficient amount of protein is ingested by healthy, young adults.

There are only few studies that have assessed the capacity of plant-derived protein to directly stimulate post-prandial muscle protein synthesis rates (Churchward–Venne et al. 2019; Pinckaers et al. 2021; Tang et al. 2009; Wilkinson et al. 2007; Yang et al. 2012). The present study is the first to evaluate the anabolic properties of corn derived protein as well as a blend of corn plus milk protein in vivo in humans. We clearly show that a deficiency in a specific EAA does not restrict the acute muscle protein synthetic response following protein ingestion. The available free EAA pool in the body resulting from protein breakdown, may be sufficient to compensate a specific EAA deficiency when protein is ingested acutely in a rested state. Especially in a real-life dietary setting where multiple plant- and animal-based proteins are ingested in mixed meals, the deficiency of a single amino acid in a specific protein source, is not likely to compromise the overall muscle anabolic response to plant-based protein ingestion in young individuals. However, whether this would also hold true for other populations such as elderly and clinical populations suffering from anabolic resistance to protein intake, remains questionable. In this regard, we have previously shown that the ingestion of 35 g wheat protein hydrolysate did not stimulate muscle protein synthesis rates in older men (Gorissen et al. 2016). Therefore, more research is warranted to establish whether the lack of specific EAA in plant-based proteins would limit its anabolic response in older compared to younger individuals, and more long term *vs* acute settings. In addition, it is important to take into consideration that the present study investigated protein isolates. Although the protein isolates have various applications (e.g.: milk formula, enriching food products with protein), corn and milk both contain only ~ 3–3.5 g protein per 100 g of food product. Evaluating the anabolic response to protein isolates is the first step to determine the anabolic potential of different protein sources. However, it is important to consider that protein quality can also be affected by other nutrients that contribute to the whole foods matrix, or processed food products. Therefore, future research will need to evaluate the anabolic response to food products containing these protein isolates, as well as whole foods and complex meals.

In conclusion, ingestion of 30 g milk protein, 30 g corn protein, or a blend of 15 g corn plus 15 g milk protein increases muscle protein synthesis rates in young, healthy males. Post-prandial muscle protein synthesis rates following the ingestion of 30 g milk protein do not differ from rates observed after ingesting 30 g corn protein or a blend providing 15 g milk plus 15 g corn protein in healthy, young males. Ingestion of a meal-like (30 g) dose of plant-derived protein can be as effective as ingesting the same amount of animal-derived protein to increase muscle protein synthesis rates in vivo in healthy, young males.

## Supplementary Information

Below is the link to the electronic supplementary material.

Supplementary file1 (DOCX 720 KB)

## Data Availability

Data described in the manuscript will be made available upon request.

## Abbreviations

AA: Amino acid
ANOVA: Analysis of variance
BCAA: Branched-chain amino acids
BMI: Body mass index
CORN: 30 g corn protein isolate
CORN + MILK: 15 g corn protein isolate + 15 g milk protein concentrate
DEXA: Dual-energy X-ray absorptiometry
EAA: Essential amino acid
FSR: Fractional synthetic rate
GC-IRMS: Gas chromatography-combustion-isotope ratio mass spectrometry
GC–MS: Gas chromatography-mass spectrometry
MILK: 30 g milk protein concentrate
MPE: Mole percent excess
MPS: Muscle protein synthesis
MyoPS: Myofibrillar protein synthesis
NEAA: Non-essential amino acid
NHANES BCA: National Health and Nutrition Examination Survey, Body composition analysis
TAA: Total amino acid
UPLC-MS: Ultra-performance liquid chromatography-mass spectrometry
