# Supplementary material for: The muscle protein synthetic response following corn protein ingestion does not differ from milk protein in healthy, young adults
Source: Amino Acids. 2024 Feb 5;56(1):8. doi: 10.1007/s00726-023-03377-z (PMC10844360; doi:10.1007/s00726-023-03377-z)
Supplement: Supplementary file 1 — Supplementary file1 (DOCX 720 KB) [file 726_2023_3377_MOESM1_ESM.docx]

Online resource belonging to:

**The muscle protein synthetic response following corn protein ingestion does not differ from milk protein in healthy, young adults**

Philippe J.M. Pinckaers^1,2^, Michelle E.G. Weijzen^2^, Lisanne H.P. Houben^2^, Antoine H. Zorenc^2^, Imre W.K. Kouw^1,2^, Lisette C.P.G.M. de Groot^1,2^, Lex. B. Verdijk^1,2^, Tim Snijders^1,2^ and Luc J.C. van Loon^1,2^

*^1^TiFN, Wageningen, The Netherlands*

*^2^NUTRIM School of Nutrition and Translational Research in Metabolism, Department of Human Biology, Maastricht University Medical Centre+, Maastricht, The Netherlands*

*^3^Division of Human Nutrition & Health, Department of Agrotechnology and Food Sciences, Wageningen University, Wageningen, The Netherlands*

**Corresponding author:** Prof. Luc J.C. van Loon, Department of Human Biology, NUTRIM School of Nutrition and Translational Research in Metabolism, Maastricht University Medical Centre+, PO box 616, 6200 MD Maastricht, The Netherlands, Tel: +31 43 388 1397, Email: [l.vanloon@maastrichtuniversity.nl](mailto:l.vanloon@maastrichtuniversity.nl)

**Statements and Declarations:** The project is organized by and executed under the auspices of TiFN (Wageningen, The Netherlands), a public – private partnership on precompetitive research in food and nutrition. Funding for this research was obtained from Tereos (Marckolsheim, France), Cargill (Minneapolis, MN, USA), Kellogg (Battle Creek, MI, USA), and the Top-sector Agri&Food.

**Short running head:** Corn versus milk protein ingestion

|  |
| --- |
| **Online Resource 1:** CONSORT flow diagram. CONSORT, Consolidated Standards of Reporting Trials. The current study was part of a larger trial with a total of 7 parallel groups (*n* = 12 per group) as indicated in the flow diagram. MILK (30 g milk protein), CORN (30 g corn protein), CORN+MILK (15 g corn protein + 15 g milk protein) |

|  |
| --- |
| **Online Resource 2:** Figure to be continued on next page |
|  |
| **Online Resource 2:** Figure to be continued on next page |
|  |
| **Online Resource 2:** Figure to be continued on next page |
|  |
| **Online Resource 2:** Figure to be continued on next page |
|  |
| **Online Resource 2:** Figure to be continued on next page |

| **Online Resource 2:** Post-prandial plasma amino concentrations during the 300 min post-prandial period following the ingestion of MILK *vs* CORN and MILK *vs* CORN+MILK. Time 0 min represents time of beverage intake. Panels B, D, F, H, J, L, N, P, R, T, V, X, Z, AB, AD, AF, AH, AJ, AL represent the 0-5 h incremental area under curve (iAUC) following protein ingestion. MILK (30 g milk protein), CORN (30 g corn protein), CORN+MILK (15 g corn protein + 15 g milk protein). Values represent means ± standard deviation; * significantly different for MILK *vs* CORN (*P*<0.05), ^#^ significantly different for MILK *vs* CORN+MILK (*P*<0.05). Repeated measures ANOVA with time as within-subject variable and interventional drink (treatment) as between-subject variable, and independent samples *t*-test were used to determine differences between groups. Values displayed below represent the *P*-values for the different panels. | | | | | | | | |
| --- | --- | --- | --- | --- | --- | --- | --- | --- |
| **Amino acid** | **2-factor repeated measures ANOVA** | | | | **Independent samples *t*-test** | | | |
|  | MILK *vs* CORN | | MILK *vs* CORN+MILK | | MILK *vs* CORN | | MILK *vs* CORN+MILK | |
| Alanine | A: | <0.001 | A: | 0.02 | B: | 0.25 | B: | 0.55 |
| Arginine | C: | <0.001 | C: | <0.01 | D: | 0.29 | D: | 0.97 |
| Asparagine | E: | <0.001 | E: | <0.01 | F: | 0.81 | F: | 0.56 |
| Cystine | G: | <0.001 | G: | 0.30 | H: | 0.90 | H: | 0.48 |
| Glutamic acid | I: | <0.001 | I: | 0.15 | J: | 0.03 | J: | 0.63 |
| Glycine | K: | <0.001 | K: | 0.05 | L: | 0.41 | L: | 0.48 |
| Histidine | M: | <0.001 | M: | 0.10 | N: | 0.08 | N: | 0.91 |
| Isoleucine | O: | <0.001 | O: | 0.04 | P: | <0.001 | P: | <0.01 |
| Ornithine | Q: | <0.001 | Q: | 0.30 | R: | 0.001 | R: | 0.31 |
| Phenylalanine | S: | <0.001 | S: | <0.001 | T: | 0.35 | T: | 0.17 |
| Proline | U: | <0.001 | U: | <0.01 | V: | <0.001 | V: | 0.76 |
| Serine | W: | <0.001 | W: | <0.01 | X: | 0.02 | X: | 0.87 |
| Threonine | Y: | <0.001 | Y: | 0.20 | Z: | <0.001 | Z: | <0.01 |
| Tryptophane | AA: | <0.001 | AA: | 0.04 | AB: | <0.001 | AB: | <0.001 |
| Tyrosine | AC: | <0.001 | AC: | <0.01 | AD: | <0.01 | AD: | 0.32 |
| Valine | AE: | <0.001 | AE: | 0.05 | AF: | <0.001 | AF: | <0.001 |
| BCAA | AG: | <0.001 | AG: | 0.001 | AH: | 0.001 | AH: | 0.56 |
| NEAA | AI: | <0.001 | AI: | 0.001 | AJ: | <0.01 | AJ: | 0.65 |
| TAA | AK: | <0.001 | AK: | <0.01 | AL: | <0.001 | AL: | 0.16 |

|  |
| --- |
|  |

| 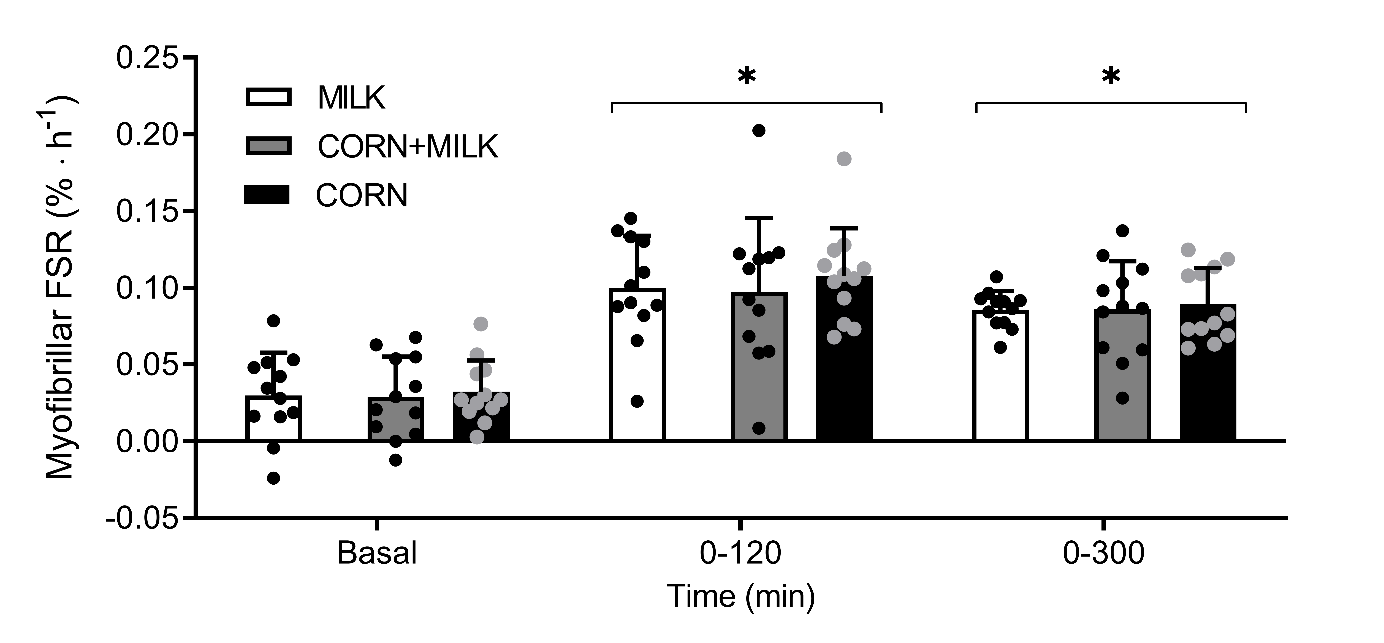 |
| --- |
| **Online Resource 3:** Myofibrillar fractional synthetic rate (FSR) determined with intra-cellular enrichments as precursor pool at different time points following ingestion of MILK *vs* CORN and MILK *vs* CORN+MILK in healthy, young males (*n*=12 per group). MILK: 30 g milk protein, CORN: 30 g corn protein, CORN+MILK: 15 g corn protein + 15 g milk protein. Bars represent means ± standard deviation, dots represent individual values. *significantly different from basal; *P*<0.05. Independent samples *t-*test: MILK *vs* CORN *P*=0.81, *P*=0.56, and *P*=0.64 for basal, 0-120, and 0-300 min, respectively. MILK *vs* CORN+MILK *P*=0.92, *P*=0.89, and *P*=0.99 for basal, 0-120, and 0-300 min, respectively. |

| **Online Resource 4:** Average 3 day dietary intake of study participants | | | | | | | | |
| --- | --- | --- | --- | --- | --- | --- | --- | --- |
|  | MILK | |  | CORN+MILK | |  | CORN | |
|  | *Mean* | *SD* |  | *Mean* | *SD* |  | *Mean* | *SD* |
| Energy (MJ∙d^-1^) | 9.3 | 2.2 |  | 7.9 | 1.7 |  | 10.2 | 2.5 |
| Carbohydrate (g∙d^-1^) | 267 | 63 |  | 216 | 63 |  | 277 | 84 |
| Fat (g∙d^-1^) | 78 | 27 |  | 69 | 27 |  | 91 | 31 |
| Protein (g∙d^-1^) | 97 | 29 |  | 80 | 29 |  | 103 | 37 |
| Energy (kJ∙kg^-1^∙d^-1^) | 131 | 26 |  | 104 | 18 |  | 133 | 30 |
| Carbohydrate (g∙kg^-1^∙d^-1^) | 3.8^#^ | 0.9 |  | 3.0 | 0.9 |  | 3.8 | 1.1 |
| Fat (g∙kg^-1^∙d^-1^) | 1.1 | 0.3 |  | 0.9 | 0.3 |  | 1.2 | 0.4 |
| Protein (g∙kg^-1^∙d^-1^) | 1.3 | 0.4 |  | 1.1 | 0.4 |  | 1.4 | 0.4 |
| Carbohydrate (% total energy) | 50 | 7 |  | 48 | 7 |  | 48 | 10 |
| Fat (% total energy) | 33 | 8 |  | 35 | 8 |  | 35 | 10 |
| Protein (% total energy) | 18 | 3 |  | 18 | 3 |  | 17 | 3 |
| Values represent mean ± standard deviation. *n*=12 per nutritional intervention group. MILK: 30 g milk protein, CORN+MILK: 15 g corn protein plus 15 g milk protein, CORN: 30 g corn protein. Independent samples *t*-test for MILK *vs* CORN and MILK *vs* CORN+MILK. ^#^significantly different for MILK *vs* CORN+MILK (*P*<0.05). 3 Day food records were analyzed using “Mijn Eetmeter” (https://mijn.voedingscentrum.nl/nl/eetmeter/), online software available from the Netherlands Nutrition Centre. | | | | | | | | |
